# Global Prospects of the Cost-Efficiency of Broiler Welfare in Middle-Segment Production Systems

**DOI:** 10.3390/ani9070473

**Published:** 2019-07-23

**Authors:** Luuk S.M. Vissers, Ingrid C. de Jong, Peter L.M. van Horne, Helmut W. Saatkamp

**Affiliations:** 1 Business Economics Group, Wageningen University, Hollandseweg 1, 6706 KN Wageningen, The Netherlands; 2 Wageningen Livestock Research, De Elst 1, 6708 WD Wageningen, The Netherlands; 3 Wageningen Economic Research, Hollandseweg 1, 6706 KN Wageningen, The Netherlands

**Keywords:** animal welfare, broiler production, cost-efficiency

## Abstract

**Simple Summary:**

Intensive broiler systems using fast-growing broiler strains at relatively high stocking density (higher than 38 kg/m^2^) are being criticised in Western countries because of risks for animal welfare. To address this criticism, alternative middle-segment production systems were introduced in North-West Europe in the 2000s. These middle-segment systems contain indoor housed slower-growing broiler strains housed at stocking densities ≤38 kg/m^2^ and claim to have increased animal welfare at a limited increase in production costs. In this study we aimed to analyse the level of animal welfare and production costs of these middle-segment production systems in different parts of the world (the Netherlands, United States and Brazil). Results show that in the Netherlands, United States and Brazil a change from conventional towards these middle-segment systems provides a considerable increase in animal welfare at a relatively small increase in production costs, i.e., has a high cost-efficiency. Overall, it can be concluded that in general middle-segment production systems entail a considerable increase in animal welfare at a relatively small increase in production costs and therefore offer good global prospects for a cost-efficient improvement of broiler welfare.

**Abstract:**

In the 2000s, the idea of a so-called middle-segment arose in North-West Europe to address the criticism on intensive broiler production systems. Middle-segment systems being indoor housing of slower-growing broiler strains at a stocking density ≤38 kg/m^2^. Previous literature showed that Dutch middle-segment systems entail a relatively large gain in animal welfare at a relatively low increase in costs, i.e., have a high cost-efficiency. The question is to what extent these findings are applicable to other countries. Therefore, the aim of this study is to gain insight in the global prospects of middle-segment systems by exploring the cost-efficiency of these systems in other parts of the world. A set of representative countries, containing the Netherlands, United States and Brazil were selected. Cost-efficiency was defined as the ratio of the change in the level of animal welfare and the change in production costs. The level of animal welfare was measured by the Welfare Quality (WQ) index score. Data was collected from literature and consulting experts. Results show that in the Netherlands, United States and Brazil a change from conventional towards a middle-segment system improves animal welfare in a cost-efficient manner (the Netherlands 9.1, United States 24.2 and Brazil 12.1). Overall, it can be concluded that in general middle-segment production systems provide a considerable increase in animal welfare at a relatively small increase in production costs and therefore offer good prospects for a cost-efficient improvement of broiler welfare.

## 1. Introduction

Broiler meat is the most consumed meat product worldwide as it is relatively cheap, low in fat as compared to other meat products and faces limited religious and cultural barriers [1]. Further increase of broiler meat production is expected due to population growth and increasing individual consumption in developing countries [2]. In the past decades, broiler meat production mainly optimized on cost-price by improving the production efficiency [3,4]. However, so-called intensive broiler production systems, using fast-growing strains at relatively high stocking densities (above 38 kg/m^2^), are being criticized in Western countries because of risks for animal welfare (e.g., EFSA Panel [5]; Bessei [6]). In addition, increasing attention for animal welfare is arising in emerging countries such as China [7]. The passing of the first global guidelines for animal welfare in 2005 by 167 countries signalled that animal welfare was no longer a concern only of prosperous nations, but had become an issue for official attention at a global level [8]. 

In North-West Europe, criticism on animal welfare in intensive broiler production systems mostly resulted in the development of organic or free-range standards for production systems, claiming higher animal welfare. However, these systems are only applied at a relatively small scale. Research showed that higher animal welfare standards in broiler production usually involve increased production costs [9]. As broiler farmers operate in a highly competitive market, the farmers’ reluctance to adopt these systems might be explained by the financial consequences of the systems, such as the need to invest in new systems and the uncertain price premium of higher animal welfare standards [10].

In the 2000s the idea of the so-called middle-segment arose in North-West Europe, which aims to have increased animal welfare at a limited increase in production costs. In this article, middle-segment systems are defined as indoor production systems using slower-growing broiler strains at stocking densities ≤38 kg/m^2^ while conventional systems are defined as indoor production systems using fast-growing broiler strains at stocking densities >38 kg/m^2^. In the Netherlands, the middle-segment Volwaard broiler production system was introduced in 2007 [11] and recently (2014–2017) Dutch retailers banned meat from broilers in conventional systems and replaced it with a middle-segment production system named the New Dutch Retail Standard (NDRS) [12]. The study of Gocsik et al. [13] showed that Dutch middle-segment systems entail a relatively large gain in animal welfare at a relatively low increase in costs, i.e., a high cost-efficiency, as compared to free-range or organic production systems. In this way, middle-segment production systems provide opportunities for changes to higher welfare production systems. The question is to what extent the findings of Gocsik et al. [13] are applicable to other countries, as production costs are determined nationally and middle-segment production systems are not existing in other countries. Therefore, global prospects in the cost-efficiency of animal welfare when changing from conventional towards alternative systems, claiming higher welfare standards, are currently missing. This insight is required to improve the adoption of alternative systems to address the criticism on animal welfare. To our knowledge this has not been investigated yet. The aim of this study is to gain insight in the global prospects of middle-segment systems by exploring the cost-efficiency of these systems in other parts of the world. To achieve this, a set of representative countries were selected for which the conventional and middle-segment production systems were identified. Consequently, the cost-efficiency of the production systems was calculated using the approach of Gocsik et al. [13].

## 2. Materials and Methods

### 2.1. Countries and Corresponding Broiler Production Systems

The following two criteria were set up to select the countries: (1) the country has a considerable contribution to global or regional broiler meat production or exports (2) the countries cover different global regions. For each country, the conventional indoor production systems, using fast-growing broiler strains, and existing alternative indoor production systems claiming higher welfare standards were identified. Outdoor broiler production systems were not considered in this study, as they require investments that limit the farmers’ flexibility to switch towards the conventional production system after a production cycle. On top of our selection, a middle-segment standardized system was designed in consultation with World Animal Protection, which is expected to provide a higher level of animal welfare than the conventional system. This production system was defined as the Global Welfare Standard (GWS). The GWS system was applied to all countries and served as a joint reference point. The system attributes of the GWS system are essentially the same in all markets, although the final weight of the broilers and the length of the growth period is set equal to the final weight of the broilers in the corresponding country.

Table 1 shows the different systems that were analysed per country. The conventional production system in the Netherlands entails the minimum standards for broiler production laid down by the European Union [14] and is mainly used for export markets [15]. The New Dutch Retail Standard (NDRS) contains the minimum standards applied by retailers and exceeds conventional standards [16]. The extensive indoor+ is a system specific to the Dutch market, produced under a national welfare label, and contains a covered veranda (indoor free-range area) and natural light entrance in the house. The conventional production system in the US includes the animal welfare guidelines for broilers established by the National Chicken Council [17]. There are certification programs in the United States which developed systems providing higher welfare standards, such as the enriched environment system of the Global Animal Partnership [18]. In Brazil, most broilers are kept in open sided houses covered with curtains along the long sides [19]. In addition, tunnel ventilated houses are increasingly used [20], which have exhaust fans at the end of the house. The open sided housing system in Brazil is considered as conventional as it is most frequently used in Brazilian broiler production [21].

### 2.2. Disaggregation of Broiler Production Systems into System Attributes

**Figure 1:**
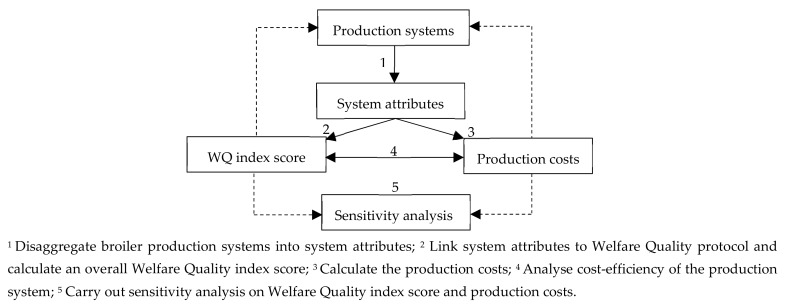
Analysis of approach (obtained from Gocsik et al. [13]).

The five-step approach developed by Gocsik et al. [13] was used to analyse the cost-efficiency of middle-segment production systems for different countries, shown in Figure 1. Below, a brief description of the method is provided (for more details see Gocsik et al. [13]).

The first step was to decompose the various production systems into system attributes which have an impact on animal welfare. Table 1 provides an overview of the system attributes of the analysed production systems. Data was collected by consulting literature, companies and research institutes.

### 2.3. Link System Attributes to Welfare Quality Protocol and Calculate an Overall Welfare Quality Index Score

#### 2.3.1. Establishment of Linkages and Weights

The Welfare Quality protocol consists of four principles, each representing a specific area of animal welfare. These principles are defined by twelve criteria and several welfare measures underlying the criteria. These welfare measures are used to calculate the Welfare Quality index score (WQ index score), which defines the overall level of animal welfare of the production system. Each criterion is expressed in a 0 to 100 value scale, in which 0 reflects the worst situation and 100 reflects the best situation of an animal unit. It should be realized that 0 is likely not achieved in practice, as it means extremely low levels of welfare that fall outside the range of what is actually measured. To determine the contribution of each system attribute to the WQ index score, the system attributes had to be linked with welfare measures. First, a yes/no indication of the link between the system attributes and welfare measures were established based on an extensive literature review. The literature review is not included in this paper but can be obtained from the authors after request. Since the set of system attributes could differ between production systems (for instance systems with/without enrichment), this could result in different linkages for some production systems. Second, weights were given to all linkages based on the relative importance of the linkage. When a welfare measure was linked to several system attributes, the relative importance of each linkage was estimated. For each linkage, a value of 1, 2 or 3 was assigned by evaluating the relative importance of the linkage. Consequently, the weight was determined by dividing the value of the linkage by the total value of the welfare measure. When a value was linked to only one attribute, a value of 1 was assigned to the linkage. In Table 2 the weights of the Dutch production systems are provided as an example.

#### 2.3.2. Calculation of WQ Index Score

The step-wise approach developed by Gocsik et al. [13] was carried out to calculate the WQ index score for each production system. This welfare score was calculated by applying the procedure of the Welfare Quality Protocol® [29]. First, for each welfare measure, a welfare score for each flock was calculated. Second, the attributional WQ score was calculated for each flock by multiplying each welfare score by the weight associated with the attribute and subsequently by summing up the weighted scores of the attribute. Third, the attributional WQ score in a production system was calculated as the mean attributional WQ score of the flocks of a given production system. Fourth, the overall WQ index score for each production system was calculated by aggregating the attributional WQ scores of the system.

Data was collected by consulting literature and contacting experts from companies and research institutes. Looking at the available data, the production systems can be distinguished into two categories. An overview of the method applied for each production system is provided in Table 3. The first category entails production systems for which extensive data as applied in the Welfare Quality Protocol® [29] was found. This held for the conventional and extensive indoor+ systems in the Netherlands and the conventional system in Brazil. In the Netherlands, data on the welfare measures were found from 76 conventional flocks and 20 extensive indoor+ flocks, assessed in 2013 (Table S1) (unpublished observations of De Jong et al. [30]). Data was found on welfare measures of the conventional (open sided) system in Brazil from Federici et al. [19] (11 flocks) and Souza et al. [31] (20 flocks). The second category entails production systems for which limited data was found. The scores for the welfare measures were estimated by expert opinion (I.C. de Jong, Wageningen Livestock Research) based on the collected data. This was the case for the NDRS system in the Netherlands, tunnel ventilated system in Brazil, the conventional and enriched environment systems in the United States and the GWS system. The scores for welfare measures of the NDRS and GWS systems were based on data of the Dutch conventional and extensive indoor+ systems. As the GWS system is a (currently) non-existing system which served as a joint reference point, it was assumed that the WQ index score of the GWS system did not differ between the analysed countries. Hence, the WQ index score of this production system was set uniformly in all countries. The welfare measures of the tunnel ventilated production system in Brazil and the conventional and enriched environment production systems in the United States were estimated by using the data of the conventional production system in Brazil. 

### 2.4. Calculation of the Production Costs and Cost-Efficiency per Broiler Production System

The production costs were calculated for each production system by using the model of Gocsik et al. [9]. Total production costs were expressed in eurocents/kg live weight to be able to compare the costs between production systems. The cost-efficiency, defined as the difference in WQ index score divided by the difference in total production costs, was analysed on production system level and on attribute level. The cost-efficiency on attribute level entails the implementation of only a single attribute of the GWS production system in the conventional production system. The following three system attributes were considered: use of a slower-growing breed, reduction of the stocking density (to 30 kg/m^2^) and providing pecking and perch enrichment (2 bales/1000 broilers, 2 m perch/1000 broilers). These attributes are selected as they are predominantly mentioned in the public discussion for improving animal welfare (e.g., Riber et al. [32]; EFSA Panel [5], Bessei [6], Averós and Estevez [33]). 

Technical input variables and prices are obtained from Van Horne [22] and by consulting literature and experts. Since price premium on feed prices of alternative systems were only available for Dutch production systems, the same price premium was used for feed prices of alternative production systems in other countries. A selection of the main variables and prices are provided in Appendix A.

### 2.5. Sensitivity Analysis of Broiler Production Systems

Since the prime interest of the study is the relative levels of animal welfare and costs, the sensitivity analysis primarily tested the robustness of the rankings. A variation in the WQ index score of 5% was used to test the robustness of the overall WQ index score. The robustness of the production costs was tested by carrying out a sensitivity analysis on two of the main cost components, i.e., feed costs and day-old chick costs. The feed prices and day-old chick prices of 2011–2015 were collected. Two scenarios were modelled: a best-case scenario and worst-case scenario. In the best-case scenario, the minimum feed and day-old chick prices of the selected time span were used, while the maximum feed and day-old chick prices were used for the worst-case scenario. Since feed prices of the selected years were only available for the conventional production systems, the prices for the alternative production systems were determined in such a way that the price premiums in the alternative production systems were the same as in the default situation. As the weights assigned to the linkages are to some extent arbitrary, a sensitivity analysis was also carried on the weights assigned to the linkages by changing these weights. For a welfare measure, one of the weights was decreased by 10%, but at least by 0.05. Additionally, the other weights of the measure were increased by a total of 10% and at least by 0.05, such that the sum of the weights of the welfare measure equalled 1. For each country, 10 rounds were simulated, where in each round the weights of one welfare measure were changed. 

## 3. Results

### 3.1. Cost-Efficiency of Production Systems

**Figure 2:**
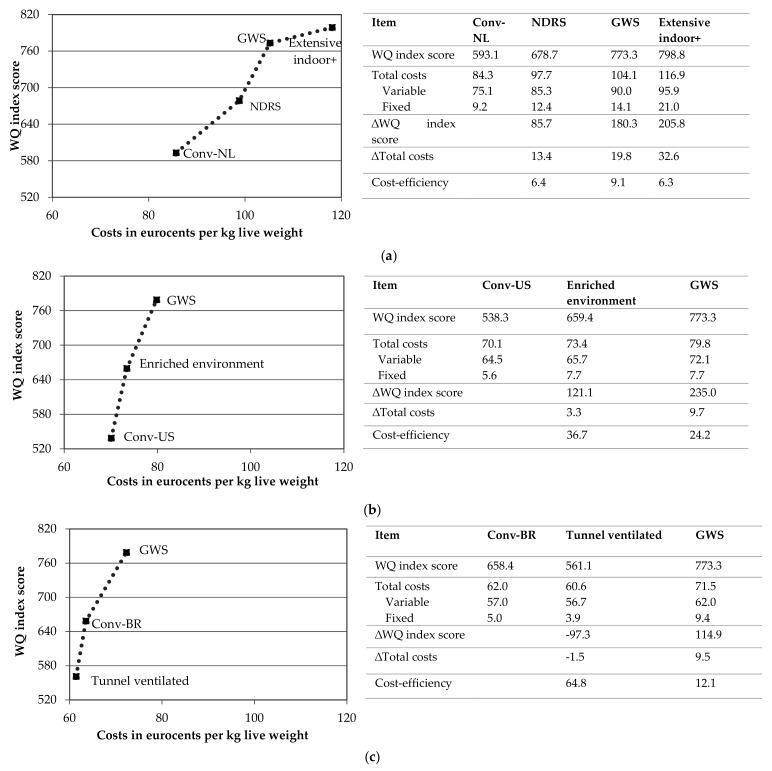
Cost-efficiency of production systems in (**a**) the Netherlands (**b**) United States and (**c**) Brazil.

In Figure 2, the results of the production systems in the Netherlands, United States and Brazil are shown. The WQ index score of the conventional systems differs between countries. In Brazil, the highest WQ index score was found (658.4), followed by the Netherlands (593.1) and United States (538.3). The results show that the level of animal welfare in the GWS system is similar in each country (similar WQ index scores), but the production costs differ which can be attributed to different prices. The results show that in each country a relatively cost-efficient improvement (defined as the ratio ∆WQ index score/∆production costs) in animal welfare can be achieved by changing from the conventional to the GWS system. In the Netherlands, this change is associated with a reasonable cost-efficiency (9.1), exceeding the cost-efficiency of changing from the conventional system to the NDRS system (6.4). Interesting to note is the decrease in cost-efficiency (6.3) when shifting beyond the GWS system, as the additional measures of the extensive indoor+ system (such as the use of a covered veranda and an additional decrease in the stocking density) further increase production costs and confer a relatively smaller additional increase in the WQ index score. In the United States, the change from conventional towards the GWS system results in a relatively cost-efficient (24.2) improvement of animal welfare. For the United States, a step in-between the conventional and GWS production system is presented, as the enriched environment production system provides a higher level of animal welfare than the conventional production system but a lower level of animal welfare than the GWS production system (+121.1 compared to +235.0 in GWS production system). An even higher cost-efficiency (36.7) can be achieved by changing from the conventional towards the enriched environment system. The lower cost-efficiency of the GWS production system can be explained by the additional measures of the GWS production system which involve a considerable increase in costs, such as the use of a slower-growing breed and a further reduction of the stocking density resulting in higher feed, housing and equipment costs. In Brazil, the shift from the conventional system towards the GWS system results in a considerable cost-efficient (12.1) improvement of animal welfare. As the tunnel ventilated production system results in a decrease of the WQ index score and production costs, the relative high cost-efficiency (64.8) reflects a considerable reduction of the WQ index score (−97.3) at only a small decrease in production total costs (−2.3%).

### 3.2. Cost-Efficiency of System Attributes

**Figure 3:**
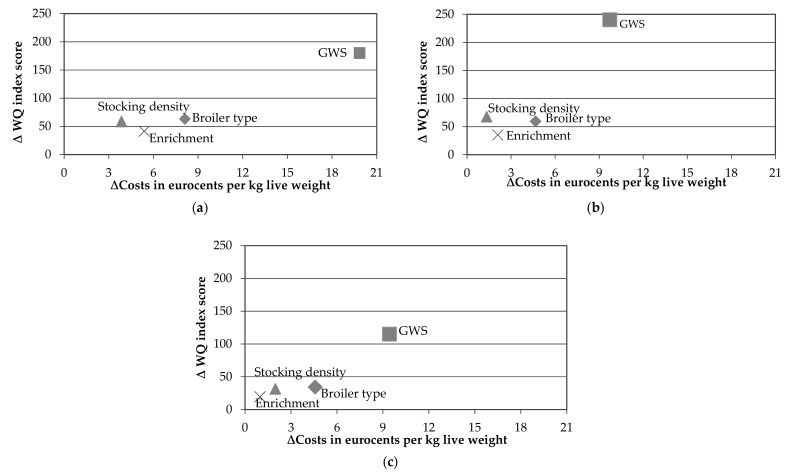
Cost-efficiency of implementing three single measures in the conventional production system in (**a**) the Netherlands (**b**) United States (**c**) Brazil.

A breakdown of the production costs per cost component is provided in Appendix B. Main contributors to total costs were feed and day-old chicks, contributing 61.6%–83.0% of total costs. When changing to a higher animal welfare system the increase in costs can be mainly attributed to the higher feed costs of slower-growing breeds. The cost-efficiency of implementing a single attribute of the GWS production system into the conventional production system was compared to the cost-efficiency of a simultaneous implementation of all system attributes in the GWS production system. The change in production costs and WQ index score when implementing one of the following three system attributes in the conventional production system are considered: use of a slower-growing breed, reduction of the stocking density (to 30kg/m^2^) and providing pecking and perch enrichment (2 bales/1000 broilers and 2 m perch/1000 broilers) in the conventional production system. Figure 3 provides the results for the Netherlands, United States and Brazil. The results only show small differences in WQ index score and production costs. In the Netherlands, a maximum difference in production costs of 4.3 eurocents per kg live weight and a maximum of 21.7 in the WQ index score was observed between the measures. A maximum difference of 3.3 eurocents per kg live weight and a maximum difference of 27.0 in the WQ index was found for the United States, while a difference of 3.6 eurocents in the production costs and 14.6 in the WQ index score was found for Brazil. 

### 3.3. Sensitivity Analysis

#### 3.3.1. Uncertainty in WQ Index Score and Fluctuations in Prices

**Figure 4:**
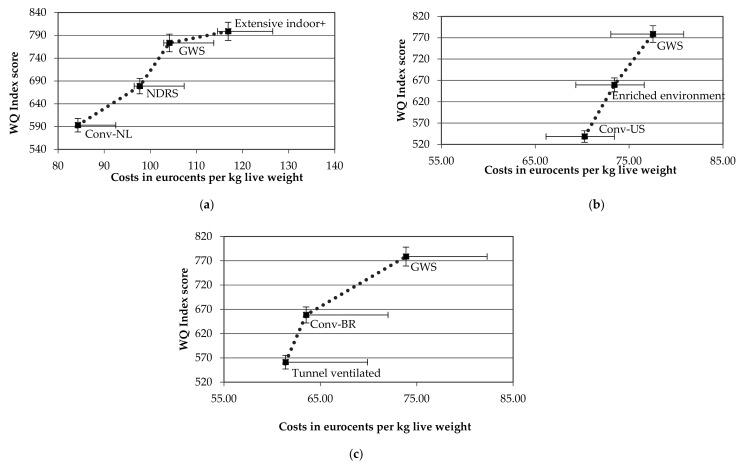
Sensitivity analysis on WQ index scores and total costs per production system (in eurocents per kg live weight) in (**a**) the Netherlands (**b**) United States and (**c**) Brazil.

The sensitivity analysis on the WQ index score and production costs for the Netherlands, Brazil and Unites States are visualized in Figure 4. The results show that the variation in WQ index score was relatively small for all production systems; overlap only existed for the GWS and extensive indoor+ production systems. With respect to the fluctuations in prices, a best-case and worst-case scenario was developed. The results show that the amplitude and thus the sensitivity of the production costs increases when changing from conventional to a middle-segment production system such as the GWS or extensive indoor+ production systems. Some overlap existed between the NDRS and GWS production systems in the Netherlands, the conventional, enriched environment and GWS production systems in the US and the tunnel ventilated and conventional production systems in Brazil. Since a change in feed and/or day-old chick prices moves all production systems in the same direction, the relative position of the production systems is not affected. For instance, an increase in feed prices negatively affects all production systems via increasing feed costs. Although increasing feed prices have a stronger negative impact on the feed costs of production systems using slower-growing breeds (caused by a higher feed conversion ratio), relative to systems using fast-growing breeds, this effect is too small to affect the relative position of the production systems. 

#### 3.3.2. Uncertainty in Assigning Weights to Linkages

To test the robustness of the weights assigned to the linkages, the impact of the change in weights on the relative contribution of the attribute to the overall WQ index score was assessed. For each production system, 10 rounds were generated for which the weights of one welfare measure were changed. This resulted in 100 new recalculated attributional WQ scores in each production system. The results show only a marginal change in the absolute and relative contribution of an attribute to the WQ index score. In the Netherlands, US and Brazil the maximum change in the relative contribution of an attribute to the WQ index score was 0.9%, 0.8% and 0.8%, respectively. The ranking of the system attributes based on the relative contribution of the system attributes remained unchanged in all rounds. The results of one round in the Netherlands, in which the weights for the welfare measure panting were changed, are provided in Appendix C.

## 4. Discussion

The aim of this study was to gain insight into the global prospects of middle-segment systems by exploring the cost-efficiency of these systems in other parts of the world. The Netherlands, United States and Brazil were selected as a set of representative countries, as these countries are important broiler producers and cover different global regions. In the current study, we focused on conventional and middle-segment production systems only, as broiler systems including an outdoor range were considered to be part of the top-market segment. For each country, the conventional production system using fast-growing broiler strains and alternative indoor production systems aiming at higher welfare standards were identified. The latter included a standardized Global Welfare System (GWS) that was modelled for each country and served as a joint reference point. Results showed that in the Netherlands, United States and Brazil a change from the conventional system towards the GWS system improves animal welfare in a cost-efficient (defined as ratio of ∆WQ index score/∆production costs) manner (the Netherlands 9.1, United States 24.2, Brazil 12.1). Interesting to note is a decrease in the cost-efficiency (6.3) when shifting beyond the GWS system (extensive indoor+ system) in the Netherlands. In the United States, a step in-between the conventional and GWS systems is present (enriched environment system) which provides an even higher cost-efficiency (36.7) than the GWS system. In Brazil, a change from conventional towards the tunnel ventilated system is highly cost-inefficient as a considerable reduction of the WQ index score occurs (−97.3) at only a small decrease in total costs (−2.3%). The cost-efficiency of the system attributes, using a slower-growing breed, a reduced stocking density (to 30 kg/m2) and providing pecking and perch enrichment (2 bales/1000 broilers, 2 m perch/1000 broilers), when changing from the conventional to the GWS production system was determined. Differences between the system attributes were very small in both the WQ index score and production costs, which imply that the attributes contribute more or less equally to the cost-efficiency of the GWS system. 

The results of the current study were obtained by applying the method developed by Gocsik et al. [13]. As for methodological issues, limitations on the calculation of animal welfare should be taken into account when interpreting the results and conclusions. Since no golden standard exists for the estimation of absolute animal welfare, any measure of animal welfare will entail some degree of arbitrariness. The Welfare Quality Protocol^®^ [29] is perceived as the most reliable method to calculate animal welfare in broiler production systems, although there are some doubts on the validity of the behavioural measures [34,35]. The Welfare Quality Protocol^®^ [29] is largely based on animal based measures, and therefore should provide a more reliable and credible picture of animal welfare than assessment of resource-based measures [36]. As different regions were included in the study, climatic differences exist among countries. Research has shown that stocking density affects broiler welfare via its interaction with ventilation and temperature [5], which should be reflected in the proportion of birds panting. This is only somewhat addressed in the Welfare Quality protocol by the proportion of birds panting at the time of the visit. The importance of stocking density may therefore be overestimated in the calculation of the WQ index score. This should be taken into consideration when interpreting the results on the cost-efficiency of the production systems and system attributes. 

The weighting of the linkages between welfare measures and system attributes is arbitrary but relative, and based on scientific literature as much as possible. However, little research was found on the magnitude of the effect of an attribute on a welfare measure. Therefore, when no literature was found, weights were estimated by expert opinion. Some caution should therefore be taken when interpreting the results. To address this uncertainty, a thorough sensitivity analysis was carried to test the robustness of the weights assigned to the linkages by altering these weights. 

The results showed that the relative contribution of an attribute to the WQ index score only changed a little (not more than 0.9%) in all countries. However, the ranking of the system attributes, based on the relative contribution of an attribute, was unaffected. This is referred to as robust. Evidence can be found in Appendix C. It should be taken into account that the weights only affect the contribution of an attribute to the WQ index score. Hence, the total WQ index score is not affected by the uncertainty of the weights. 

Another limitation was the scarcity of data available on welfare measures collected according to the Welfare Quality Protocol^®^ [29]. An extensive database for the Netherlands, which contained data of the Dutch conventional (76 flocks) and extensive indoor+ production (20 flocks) systems [30], and some data for the Brazilian conventional production system (31 flocks) [19,31] were found. Because the data was detailed and no other data was available, this data was used as a reference for derivation of animal welfare estimates of the NDRS, GWS, US conventional, enriched environment and tunnel ventilated systems using expert opinion (I.C. de Jong, Wageningen Livestock Research). Uncertainty associated with these estimations should be taken into account. To address the uncertainty associated with the data, the robustness of the overall WQ index score and production costs were tested. The results showed that although there was some variation in the WQ index score, the relative positions of the production systems were unaffected. The sensitivity analysis on production costs showed a relatively large variation in production costs which was mainly caused by the change in feed prices. In addition, the results showed that the amplitude and thus the sensitivity of the production costs increased when shifting from the conventional production system towards a middle-segment production system (GWS or extensive indoor+ system), which can be explained by the higher feed conversion ratio of a slower-growing breed. Although changing feed prices have a stronger impact on production systems with a slower-growing breed (via a higher feed conversion ratio) than production systems with a fast-growing breed, this effect is relatively small. Therefore, the relative positions of the production systems in terms of production costs were robust. 

## 5. Conclusions

As the sensitivity analysis showed that the ranking of the production systems were robust in all countries, we can conclude that middle-segment production systems offer good global prospects for improving broiler welfare at a relatively small increase in costs, i.e., at a high cost-efficiency. This conclusion is comparable to the conclusion of Gocsik et al. [13] for the Netherlands, which showed that Dutch middle-segment systems are relatively cost-efficient. 

Assuming the high cost-efficiency of improving animal welfare by changing towards middle-segment production systems, the subsequent question is how to implement these systems. It should be taken into account that only small increases in costs per animal can have a large impact on farmers’ profits. Given the high competitiveness of the broiler sector, farmers may be reluctant to implement production systems entailing higher costs. Farmers are only willing to adopt a middle-segment production system if the additional costs are covered by a price premium, which will result in a higher consumer price. Especially in developing countries, this price increase should be taken into consideration to allow low-income consumers to buy animal proteins. The study of Mulder et al. [16] showed that there is a willingness of Dutch consumers to pay for higher broiler welfare. However, active involvement of other stakeholders, i.e., retail, is also required to successfully impose middle-segment systems [12]. As the level of animal welfare in conventional systems and the costs are country specific, the starting situation and the obstacles for improving animal welfare are different for each country. Finally, it can be concluded that in general middle-segment production systems provide a considerable increase in animal welfare at a relatively small increase in production costs and therefore offer good prospects for a cost-efficient improvement of broiler welfare. 

## Figures and Tables

**Table 1:** Overview of the system attributes of the broiler production systems.

**Production System**	**System Attributes**									
	Broiler Type	Length Growth Period (Days)	Live Weight (Grams)	Out Door	Stocking Density (kg/m^2^)	Natural Light	Enrichment (Grains, Straw Bales, Perches)	Light Intensity (lux)	Dark Period (Hours/Day)	Floor Type
Applied in all countries										
Global Welfare Standard	Slower-growing	^1^	^1^	No	30	Yes	2 bales/1000 broilers 2m perch/1000 broilers	20	6	Concrete with litter [22]
The Netherlands										
Conv-NL	Fast-growing [13]	38 [22]	2300 [22]	No	42 [13]	No [13]	No [13]	20 [14]	6 [13]	Concrete with litter [22]
New Dutch Retail Standard	Slower-growing [23]	47 [23]	2400 [24]	No	38 [23]	No [23]	2g/broiler 1 bale/1000 broilers [23]	20 [14]	6 [13]	Concrete with litter [22]
Extensive Indoor+	Slower-growing [25]	51 [22]	2300 [13]	Cov. veranda ≥ 20% total area [25]	25 [25]	Yes [25]	2g/broiler 1 bale/1000 broilers [25]	20 [25]	8 [25]	Concrete with litter [22]
United States										
Conv-US	Fast-growing [22]	46 [22]	2700 [22]	No	37 [22]	No [17]	No [17]	5 [26]	4 [17]	Concrete with litter [17]
Enriched environment	Fast-growing [22]	46 [22]	2700 [22]	No	32 [18]	No [18]	Grains 1% of diet 1 bale/70m2 [18]	50 [18]	8 [18]	Concrete with litter [18]
Brazil										
Conv-BR	Fast-growing [22]	44 [22]	2600 [22]	No	34 [22]	Yes [19]	Min 60% grains in feed [21]	Natural light +5 lux [19]	8 [21]	Dirt with litter [27]
Tunnel ventilated	Fast-growing [22]	44 [22]	2600 [22]	No	38 [22]	No [21]	Min 60% grains in feed [21]	5 [28]	8 [19]	Dirt with litter [27]

^1^ Live weight (length growth period) is dependent on final weight of broilers in corresponding country. The Netherlands 2400g (49 days), United States 2700g (55 days), Brazil 2600g (53 days).

**Table 2:** Matrix showing weights between the welfare measures and system attributes of the Dutch broiler production systems.

**Welfare Measures**	**System Attributes**										
	A1. Broiler type	A2. Length growth period	A3. Weight at delivery	A4.1 Enrichment – (straw) bales	A5. % Grain in feed	A6. Stocking density	A7. Natural light	A8. Length dark period	A9. Flock size	A10. Concrete litter floor	A11. Light intensity
3.1: Plumage cleanliness		0.25		0.25		0.25				0.25
3.2: Litter quality		0.17				0.33	0.17	0.17		0.17
4.1: Panting	0.50					0.50				
5.1: Stocking density						1				
6.1: Lameness	0.13	0.13	0.13	0.13		0.25	0.13	0.13		
6.2: Hock burn	0.17	0.17	0.17			0.17		0.17			0.17
6.3: Foot pad dermatitis	0.22	0.11		0.11	0.11			0.11		0.22	0.11
6.4: Breast blister	1									
7.1: On farm mortality	0.50							0.50		
7.3: Ascites	0.67							0.33		
7.5: Septicaemia										
7.7: Pericarditis										
11.1: ADT ^1^								0.67	0.33	
12.1: QBA ^2^				0.25		0.13	0.13	0.13	0.13	0.13	0.13

^1^ Avoidance Distance Test; ^2^ Qualitative Behaviour Assessment; Legend: 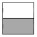 Linkage established on literature (white); Linkage based on expert opinion I.C. de Jong, Wageningen Livestock Research (grey).

**Table 3:** Method applied for each production system.

Country	Broiler Production System	Method
Applied in all countries	GWS	Expert estimation based on data of the Netherlands
Netherlands	Conv-NL	Data [30]
	NDRS	Expert estimation based on data of the Netherlands
	Extensive indoor+	Data [30]
United States	Conv-US	Expert estimation based on data of Brazil
	Enriched environment	Expert estimation based on data of Brazil
Brazil	Conv-BR	Data [19,31]
	Tunnel ventilated	Expert estimation based on data of Brazil

## Supplementary Materials

The following are available online at https://www.mdpi.com/2076-2615/9/7/473/s1, Table S1: Technical data of the Dutch broiler production systems collected according to the WQ protocol [29].

## Appendix A

**Table A1:** Overview of main technical inputs and prices used in the economic model of Gocsik et al. [9].

**Country**	**Production System**	***Item***					
		Live weight	Feed conversion rate (g/g)	Mortality (%)	Feed price (€/100kg)	Day-old chick price (eurocents/bird)	Labour (€/hour)
The Netherlands	Conv-NL	2300 [22]	1.61 [22]	3.5 [22]	32.7 [22]	31.5 [22]	24.0 [22]
	NDRS	2400 [24]	1.88 [24]	2.5 [24]	32.7 [22]	33.5 [22]	24.0 [22]
	Extensive Indoor+	2300 [22]	2.09 [22]	2.5 [13]	32.7 [22]	33.5 [22]	24.0 [22]
	GWS	2400	1.88 [24]	2.5 [24]	32.7 [22]	33.5 [22]	24.0 [22]
United States	Conv-US	2700 [22]	1.86 [22]	4.5 [22]	26.3 [22]	25.1 [22]	13.5 [22]
	Enriched environment	2700 [22]	1.86 [22]	4.5 [22]	26.3 [22]	25.1 [22]	13.5 [22]
	GWS	2700 [22]	2.03 [22]	2.5 [24]	26.3 [22]	27.1 ^1^	13.5 [22]
Brazil	Conv-BR	2600 [22]	1.79 [22]	5.2 [19]	23.6 [22]	23.6 [22]	3.0 [22]
	Tunnel ventilated	2600 [22]	1.79 [22]	4.0 [22]	23.6 [22]	23.6 [22]	3.0 [22]
	GWS	2600 [22]	1.96 [22]	2.5 [24]	23.6 [22]	25.6 ^1^	3.0 [22]

^1^ Price premium of slower-grower was assumed to be similar to the price premium in the Netherlands.

## Appendix B

**Table A2:** Breakdown of production costs per cost component (in eurocents per kg live weight).

**Country**	**Production System**	**Variable Costs**							**Fixed Costs**				
		Feed	Day-old chicks	Animal health	Litter	Grain and straw	Catching	Other variable costs ^1^	General	Labour	Housing	Equipment	Total
The Netherlands	Conv-NL	52.7	13.7	2.0	0.4	0	2.1	4.3	0.8	2.7	3.6	2.1	84.3
	NDRS	61.5	14.0	1.5	0.6	0.6	2.0	5.0	0.8	3.5	5.1	3.1	97.7
	GWS	61.5	14.0	1.5	0.6	1.4	2.0	5.7	0.8	6.0	6.4	4.3	104.1
	Extensive indoor+	68.3	14.6	1.6	1.0	0.8	2.1	7.6	0.7	4.8	9.7	5.8	116.9
United States	Conv-US	48.9	9.3	1.7	0.2	0	0.2	3.6	0.7	1.5	2.3	1.7	70.1
	Enriched environment	48.9	9.3	1.7	0.2	0.6	0.2	4.7	0.7	2.3	2.7	2.1	73.4
	GWS	53.3	10.0	1.3	0.2	0.7	0.9	5.2	0.7	1.8	3.1	2.6	79.8
Brazil	Tunnel ventilated	42.2	9.1	1.2	0	0	0.2	1.5	0.4	1.5	2.0	2.5	60.6
	Conv-BR	42.2	9.1	1.2	0	0	0.2	2.9	0.4	2.0	1.8	2.2	62.0
	GWS	46.3	9.9	0.9	0.2	0.7	0.2	3.8	0.4	2.3	2.8	4.0	71.5

^1^ Includes: heating, water, electricity, dead bird disposal and other.

## Appendix C

**Table A3:** Sensitivity analysis results on changing weights for the system attributes of the welfare measure panting in the Netherlands (changes are depicted in bold).

**Production System**	**System Attribute**	**WQ Scores per Attribute**		**Relative Contribution to WQ Score**	
		Default weights broiler type (=0.50), stocking density (=0.50)	With new weightsbroiler type (=0.55), stocking density (=0.45)	Default weights broiler type (=0.50), stocking density (=0.50)	New weights broiler type (=0.55), stocking density (=0.45)
Conv-NL	Broiler type	**229.1**	**233.6**	**38.6** **%**	**39.4** **%**
	Length growth period	19.6	19.6	3.3%	3.3%
	Weight at delivery	7.5	7.5	1.3%	1.3%
	Straw bale enrichment	15.8	15.8	2.7%	2.7%
	Perch enrichment	-	-	0.0%	0.0%
	Grain in feed	3.9	3.9	0.7%	0.7%
	Stocking density	**102.2**	**97.7**	**17.2** **%**	**16.5** **%**
	Natural light	8.7	8.7	1.5%	1.5%
	Length dark period	137.2	137.2	23.1%	23.1%
	Flock size	35.9	35.9	6.1%	6.1%
	Litter floor	19.4	19.4	3.3%	3.3%
	Light intensity	13.7	13.7	2.3%	2.3%
	WQ index score	593.0	593.0	100.0%	100.0%
NDRS	Broiler type	**260.2**	**264.8**	**38.3** **%**	**39.0** **%**
	Length growth period	30.6	30.6	4.5%	4.5%
	Weight at delivery	13.1	13.1	1.9%	1.9%
	Straw bale enrichment	21.2	21.2	3.1%	3.1%
	Perch enrichment	-	-	0.0%	0.0%
	grain in feed	6.4	6.4	0.9%	0.9%
	Stocking density	**115.1**	**110.4**	**17.0** **%**	**16.3** **%**
	Natural light	15.4	15.4	2.3%	2.3%
	Length dark period	142.1	142.1	20.9%	20.9%
	Flock size	25.3	25.3	3.7%	3.7%
	Litter floor	28.3	28.3	4.2%	4.2%
	Light intensity	21.0	21.0	3.1%	3.1%
	WQ index score	678.7	678.7	100.0%	100.0%
Extensive indoor+	Broiler type	**269.1**	**273.9**	**33.7** **%**	**34.3** **%**
	Length growth period	41.5	41.5	5.2%	5.2%
	Weight at delivery	18.7	18.7	2.3%	2.3%
	Straw bale enrichment	26.5	26.5	3.3%	3.3%
	Perch enrichment	-	-	0.0%	0.0%
	grain in feed	9.0	9.0	1.1%	1.1%
	Stocking density	**164.6**	**159.9**	**20.6** **%**	**20** **.0%**
	Natural light	22.2	22.2	2.8%	2.8%
	Length dark period	155.2	155.2	19.4%	19.4%
	Flock size	26.3	26.3	3.3%	3.3%
	Litter floor	37.3	37.3	4.7%	4.7%
	Light intensity	28.4	28.4	3.6%	3.6%
	WQ index score	798.8	798.8	100.0%	100.0%
GWS	Broiler type	**220.3**	**225.0**	**28.5** **%**	**29.1** **%**
	Length growth period	36.9	36.9	4.8%	4.8%
	Weight at delivery	15.4	15.4	2.0%	2.0%
	Straw bale enrichment	20.0	20.0	2.6%	2.6%
	Perch enrichment	94.2	94.2	12.2%	12.2%
	grain in feed	8.1	8.1	1.0%	1.0%
	Stocking density	**129** **.2**	**124.4**	**16** **.7%**	**16.1** **%**
	Natural light	18.9	18.9	2.4%	2.4%
	Length dark period	149.0	149.0	19.3%	19.3%
	Flock size	24.4	24.4	3.2%	3.2%
	Litter floor	33.2	33.2	4.3%	4.3%
	Light intensity	23.6	23.6	3.1%	3.1%
	WQ index score	773.3	773.3	100.0%	100.0%
